# Supplementary material for: Reported adverse events following COVID-19 vaccination in gynecologic cancer patients in Thailand: A descriptive study
Source: PLoS One. 2026 Feb 27;21(2):e0342303. doi: 10.1371/journal.pone.0342303 (PMC12948105; doi:10.1371/journal.pone.0342303)
Supplement: S1 Table — Adverse events following immunization (AEFIs) are summarized by COVID-19 vaccine type. Data are presented as number (percentage). Local AEFIs include injection-site reactions, and systemic AEFIs include generalized symptoms. “No side effects” refers to participants reporting no adverse events following vaccination. (DOCX) [file pone.0342303.s001.docx]

S1 Table. Adverse Events Following Immunization (AEFIs) by Vaccine Type

| **Vaccine Type** | **Total** | **Total AEs (n)** | **Local AEs (%)** | **Systemic AEs (n)** | **No Side Effects (n)** |
| --- | --- | --- | --- | --- | --- |
| **Sinovac** | 19 | 19 (100%) | 14 (73.7%) | 5 (26.3%) | 0 (0%) |
| **AstraZeneca** | 520 | 404 (77.7%) | 333 (64.0%) | 71 (13.7%) | 116 (22.3%) |
| **Pfizer** | 160 | 145 (90.6%) | 109 (68.1%) | 36 (22.5%) | 15 (9.4%) |
| **Moderna** | 124 | 103 (83.1%) | 85 (68.6%) | 18 (14.5%) | 21 (16.9%) |

Adverse events following immunization (AEFIs) are summarized by COVID-19 vaccine type. Data are presented as number (percentage). Local AEFIs include injection-site reactions, and systemic AEFIs include generalized symptoms. “No side effects” refers to participants reporting no adverse events following vaccination.
