# Supplementary material for: Reported adverse events following COVID-19 vaccination in gynecologic cancer patients in Thailand: A descriptive study
Source: PLoS One. 2026 Feb 27;21(2):e0342303. doi: 10.1371/journal.pone.0342303 (PMC12948105; doi:10.1371/journal.pone.0342303)
Supplement: S2 Table — Multivariable logistic regression analysis was performed to identify factors associated with the occurrence of any-grade adverse events following immunization after the first dose of COVID-19 vaccine. Results are presented as adjusted odds ratios (ORs) with 95% confidence intervals (CIs). P-values <0.05 were considered statistically significant. BMI categories were defined according to Asia-Pacific criteria. (DOCX) [file pone.0342303.s002.docx]

**S2 Table. Fully adjusted logistic regression of factors associated with any grade adverse events after the 1st dose of COVID-19 vaccine**

| **Characteristic** | **Adjusted OR**  **(95% CI)** | **P-value** |
| --- | --- | --- |
| **Age** |  |  |
| 18 to <60 years  ≥ 60 years | 2.62 (1.25-5.47)  Reference | 0.01  1 |
| **Body mass index (kg/m^2^)**^a^ |  | 0.243 |
| Underweight (< 18.5)  Normal (18.5 – 22.9)  Overeweight/Obsese (≥23.0) | 3.72 (0.79-17.41)  Reference  1.05 (0.56-1.98) | 0.094  1  0.878 |
| **Comorbidities** |  |  |
| No  Yes | Reference  1.24 (0.65-2.38) | 1  0.51 |
| **Type of cancer** |  | 0.36 |
| Cervical  Ovary/Peritoneum/Fallopian tube  Uterine  Other gynecologic cancer | 0.49 (0.21-1.19)  Reference  0.87 (0.41-1.83)  0.44 (0.12-1.69) | 0.11  1  0.71  0.23 |
| **FIGO stage** |  | 0.34 |
| I  II  III  IV and Recurrent disease | Reference  0.85 (0.31-2.35)  0.55 (0.26-1.16)  1.25 (0.35-4.40) | 1  0.75  0.12  0.73 |
| **ECOG Performance status** |  | 0.89 |
| 0  1  ≥ 2 | Reference  1.16 (0.53-2.51)  1.29 (0.41-3.59) | 1  0.70  0.62 |
| **Current status of treatment** |  | 0.92 |
| Surveillance/Palliative treatment  Ongoing treatment  Chemotherapy  Radiation and others | Reference  1.18 (0.45-3.08)  1.18 (0.39-3.59) | 1  0.73  0.77 |

Multivariable logistic regression analysis was performed to identify factors associated with the occurrence of any-grade adverse events following immunization after the first dose of COVID-19 vaccine. Results are presented as adjusted odds ratios (ORs) with 95% confidence intervals (CIs). P-values <0.05 were considered statistically significant. BMI categories were defined according to Asia-Pacific criteria.

ECOG, Eastern Cooperative Oncology Group; FIGO, International Federation of Gynecology and Obstetrics.
