# Supplementary material for: Reported adverse events following COVID-19 vaccination in gynecologic cancer patients in Thailand: A descriptive study
Source: PLoS One. 2026 Feb 27;21(2):e0342303. doi: 10.1371/journal.pone.0342303 (PMC12948105; doi:10.1371/journal.pone.0342303)
Supplement: S1 File — This file contains the structured interview questionnaire used to collect patient information, COVID-19 vaccination history, and adverse events following immunization. Adverse events were assessed for local and systemic reactions occurring within 7 days after each vaccine dose, as well as delayed adverse events up to 30 days post-vaccination. Severity of symptoms was graded as mild, moderate, or severe based on patient self-report. (DOCX) [file pone.0342303.s003.docx]

# S1 File. Interview Questionnaire for Adverse Events after COVID-19 Vaccination

## Section A. Patient Information

1. Patient ID: ________

2. Age: ________ years

3. Cancer diagnosis: __________________________

4. FIGO stage: ________

5. Current treatment status: ☐ Surveillance ☐ Palliative ☐ Chemotherapy ☐ Radiotherapy ☐ Chemoradiation ☐ Targeted therapy ☐ Immunotherapy ☐ Hormonal therapy

## Section B. Vaccination History

1. Vaccine type (first dose): ☐ AstraZeneca ☐ Sinovac ☐ Moderna ☐ Pfizer ☐ Other: _______

2. Date of vaccination (first dose): ____/____/2021

3. Vaccine type (second dose): _____________________

4. Date of vaccination (second dose): ____/____/2021

5. Subsequent doses (if any): _____________________

## Section C. Adverse Events within 7 Days of Vaccination

*(Repeat questions for Dose 1, 2, 3, and 4)*

### Local reactions

1. Did you experience injection site pain, redness, or swelling?
 - ☐ Yes ☐ No
 - If yes: duration ___ days, severity: ☐ Mild ☐ Moderate ☐ Severe

### Systemic reactions

2. Fever
 - Did you experience fever? ☐ Yes ☐ No
 - If yes: Did you measure your temperature?
 - ☐ Yes → Highest recorded: ____ °C
 - ☐ No → How would you describe it?
 - ☐ Low-grade / mild (felt warm, did not limit daily activity)
 - ☐ Moderate (interfered with some activities, needed rest/medication)
 - ☐ Severe (required hospital visit/medical care)
 - Duration: ___ days

3. Headache ☐ Yes ☐ No → Severity: ☐ Mild ☐ Moderate ☐ Severe

4. Fatigue ☐ Yes ☐ No → Severity: ☐ Mild ☐ Moderate ☐ Severe

5. Myalgia (muscle pain) ☐ Yes ☐ No → Severity: ☐ Mild ☐ Moderate ☐ Severe

6. Nausea/Vomiting ☐ Yes ☐ No → Severity: ☐ Mild ☐ Moderate ☐ Severe

7. Diarrhea ☐ Yes ☐ No → Severity: ☐ Mild ☐ Moderate ☐ Severe

8. Rash ☐ Yes ☐ No → Severity: ☐ Mild ☐ Moderate ☐ Severe

9. Lymphadenopathy (swollen glands) ☐ Yes ☐ No

## Section D. Adverse Events after 7 Days (up to 30 Days Post-vaccination)

1. Any new or persistent symptoms? ☐ Yes ☐ No
 - If yes, specify: ____________________________
 - Duration: ___ days

## Section E. COVID-19 Infection

1. Did you test positive for COVID-19 at least 14 days after vaccination? ☐ Yes ☐ No
 - If yes: ☐ ATK positive confirmed by PCR ☐ PCR positive only
 - Date of diagnosis: ____/____/2021

## Section F. Additional Notes

(Open-ended for patients to describe other symptoms or experiences.)

This file contains the structured interview questionnaire used to collect patient information, COVID-19 vaccination history, and adverse events following immunization. Adverse events were assessed for local and systemic reactions occurring within 7 days after each vaccine dose, as well as delayed adverse events up to 30 days post-vaccination. Severity of symptoms was graded as mild, moderate, or severe based on patient self-report.
