# Supplementary material for: Reported adverse events following COVID-19 vaccination in gynecologic cancer patients in Thailand: A descriptive study
Source: PLoS One. 2026 Feb 27;21(2):e0342303. doi: 10.1371/journal.pone.0342303 (PMC12948105; doi:10.1371/journal.pone.0342303)
Supplement: Attachment — Submitted filename: NP-Response to Reviewers.docx [file pone.0342303.s005.docx]

**Point-by-point Response to Reviewers**

**Response to Reviewer 1**

Comment 1: What is the rationale for specifically looking at AEFIs in gynecologic cancer patients? Do they simply represent a subset of immunocompromised patients?

Response: We thank the reviewer for this important point. Gynecologic cancer patients, while being a subset of immunocompromised individuals, have distinct characteristics including gender specificity, treatment regimens, and regional health behaviors that could influence vaccine response. Additionally, in our setting, gynecologic oncology patients were among the prioritized groups for early COVID-19 vaccination. This subgroup analysis adds specific insights to guide clinical counseling in similar populations. This rationale has now been added to the Introduction on page 5, lines 89 -94.

Comment 2: The manuscript makes definitive claims that are not possible by the analysis. For example, “This study determines the safety of the COVID-19 vaccine in vaccinated patients with 10 gynecologic cancer by assessing side effects and side effect severity.” However, this is not vaccine safety study but rather a descriptive study of reported AEFIs in vaccinated patients. The authors should be careful with language that can be interpreted causally. It would be more accurate to say that “this study evaluates the prevalence of reported adverse events following receipt of COVID-19 vaccine.”

Response: We fully agree that our study design is descriptive and observational in nature and does not allow for causal inference regarding vaccine safety. In response, we have revised the manuscript to remove any language that implies causality. Specifically, the phrase has been changed to: “This study evaluates the prevalence and severity of reported adverse events following COVID-19 vaccination in patients with gynecologic cancer.” (page 6, line 96)

We have reviewed the entire manuscript to ensure similar adjustments are made wherever appropriate to accurately reflect the descriptive scope of the study.

- In the Abstract (page 2, line 20) and throughout the manuscript, we have replaced language that implied definitive conclusions about vaccine safety. The phrase “this study determines the safety...” has been revised to “this study evaluates the prevalence and characteristics of reported adverse events following COVID-19 vaccines.”
- Similar adjustments have been made in the Results, Discussion, and Conclusion sections to avoid any causal implications.

Comment 3: Of the 846 patients with gynecologic cancers, 294 were eligible for analysis. Please provide a flowchart of patient inclusion and exclusion, reasons for exclusion, information on excluded patients if available, and address possible selection bias. Also clarify the handling of self-reported AEFIs, recall bias due to app use.

Response: From the initial 846 patients identified with gynecologic cancers during the study period, 294 patients were included in the final analysis. The remaining patients were excluded for the following main reasons (page 8, lines 145 – 147):

1) A substantial proportion could not be contacted despite multiple attempts;
2) Some patients received their COVID-19 vaccination outside our hospital and their vaccination or adverse event records were unavailable
3) A small number were under 18 years of age at the time of vaccination, and thus did not meet eligibility criteria.

Due to the retrospective nature of the study and the lack of complete documentation for excluded patients, we were unable to quantify each exclusion category precisely.

As we did not retain detailed clinical data on the excluded patients, we were unable to compare them directly with the included sample. However, we recognize that this limitation may introduce selection bias. For example, patients with more advanced disease or more intensive treatment may have been harder to reach or less likely to participate, potentially skewing the final sample toward healthier individuals. We have now expanded our discussion of this potential bias and its possible impact on generalizability and interpretation of adverse event rates (page 21, lines 298 – 301).

Comment 4: The authors should expand the limitations section:

**1)** Self-reported AEFIs may be prone to misclassification. Were patients asked to report measured fever or just symptoms?
**2)** Clarify recall bias—does app usage improve recall compared to those without entries?

Response: We have clarified in the manuscript that adverse events were self-reported based on symptoms without objective confirmation. We also specified that recall bias may have occurred, as patients who recorded symptoms in the Mohprompt app likely had better recall than those relying on memory alone. Both limitations are now explicitly stated in the revised Discussion section (page 21, line 301 – page 22, line 304).

Comment 5: Since the majority of patients in the study are in remission, with lower ECOG performance status scores and FIGO stage, can the authors comment on the possibility that the patients included in their study are healthier and the potential impact on their results? Particularly since the authors observe lower odds of adverse events in people with comorbidities.

Response: We acknowledge that our study population, largely composed of patients in remission with lower ECOG scores and earlier FIGO stages, may represent a healthier subset. This may have contributed to the lower incidence and severity of adverse events observed. Additionally, the association between comorbidities and lower odds of adverse events may reflect underreporting or altered immune responses in patients with chronic illnesses. We have addressed these considerations in the revised Discussion (page 20, lines 298 – 301).

Comment 6: For the analyses examining any grade AEFI by treatment status in Table 4, can the authors specify the comparisons for the Fisher’s exact tests? How did the authors handle categories with fewer than two observations?

Response: We have clarified in the revised Methods section that patients were categorized into two groups: active cancer treatment versus surveillance or palliative care (page 8, lines 133 - 137). Fisher’s exact test was used to compare adverse event rates between these two broader groups. We did not perform separate analyses for individual treatment subcategories with fewer than two patients to avoid unstable statistical estimation.

Comment 7: The authors mention using Chi-square and Fisher’s exact test in the methods section but it looks like they only report results from Fisher’s exact tests. Did the authors use both for the same analyses or different methods? If the former, why?

Response: Although both tests were initially considered, all reported p-values are from Fisher’s exact tests to ensure consistency and accuracy given the small cell sizes in several subgroups. This has now been clarified in the Methods section ( page 8, lines 133 - 137).

Comment 8: Do the authors have information on timing between the doses? It would be informative to know the mean or median time between 1st/2nd/3rd/4th doses in this study population.

Response: we have now included information on the timing between vaccine doses. The median intervals between doses were as follows:

Dose 1 to 2: 11.6 weeks (SD 3.3)

Dose 2 to 3: 20.5 weeks (SD 11.7)

Dose 3 to 4: 20.3 weeks (SD 6.8)

These data have been added to the Results section (page 11, lines 172 - 175).

Comment 9: Based on Table 2, it seems that there is a bimodal distribution of vaccine types across doses (Sinovac/AstraZeneca for 1st–2nd doses and Moderna/Pfizer for 3rd–4th doses). Did the authors consider analyzing AEFIs by specific vaccine type rather than collapsing by dose?

Response: we have conducted a stratified analysis of adverse events by vaccine type. The findings show variation in the frequency of local and systemic side effects across vaccine platforms. These results are included in Supplementary Table 1.

Comment10: Line 18 – There is a typo in this sentence: “between January 2020 and August 2021 were reviewed was a descriptive…”

Response: We have corrected the sentence for clarity (page 6, lines 106-107).

**Response to Reviewer 2**

Comment 1: Abstract: Minor improvements could include briefly mentioning the statistical methods.

Response: We have revised the Abstract to briefly mention the use of logistic regression and Fisher’s exact test to reinforce the study’s methodological rigor. (page 2, lines 27-30)

Comment 2: A stronger emphasis on how this study advances current knowledge would further highlight its novelty

Response: We have revised the Introduction to highlight the novelty of our study, emphasizing that data on vaccine tolerability specific to gynecologic cancer patients are limited, and this study addresses that gap (page 5 line 89 – page 6 line 100).

Comment 3: The authors should discuss how representative their patient population is compared to the broader population of gynecologic cancer patients in Thailand.

Response: We clarified that the study cohort from a tertiary center may not fully represent all gynecologic cancer patients in Thailand (page 22, lines 308 - 313).

Comment 4: Clarification is needed on the timeline of vaccine administration in this setting

Response: The vaccination timeline was explained on page 6, lines 106 – 108. The interval between the vaccinations was added to the results on page 11, lines 172 – 175.

Comment 5: Details on the interview process should be provided.

Response: We added that structured telephone interviews were conducted using a standardized questionnaire by trained oncology staff (page 7, lines 116 – 121).

Comment 6: Information on the response rate and how missing or incomplete data were handled

Response: From the initial 846 patients identified with gynecologic cancers during the study period, 294 patients were included in the final analysis. The remaining patients were excluded for the following main reasons (page 8, lines 245 – 147).

Comment 7: The manuscript would benefit from more explicit descriptions regarding whether events were solicited or unsolicited and the exact time points

Response: We have clarified the definitions and time frames in the revised manuscript. Specifically, solicited adverse events were recorded within 7 days post-vaccination, while unsolicited events were recorded from day 8 to 30 days post-vaccination (page 7, lines 126 – 129).

Comment 8: A stratified analysis by vaccine type

Response: We performed an exploratory analysis stratified by vaccine type (inactivated, viral vector, mRNA), included in the Supplementary Table 1.

Comment 9: The analysis should address potential confounding factors such as comorbidities, ECOG performance status, and specific cancer subtypes.

Response: We acknowledge that while age was adjusted for in the logistic regression model, other potential confounders—such as comorbidities, ECOG performance status, and cancer subtypes—were not fully accounted for. This limitation has now been noted in the revised Discussion section (page 22, lines 304 – 306).

Comment 10: Further subgroup analyses would help clarify whether treatment status truly has no impact on vaccine safety.

Response: Table 4 has been revised to clearly compare adverse events between patients receiving active treatment and those under surveillance or palliative care.

Comment 11: The clinical relevance of predominantly mild events should be discussed in more depth.

Response: We discuss in more depth regarding the clinical relevance of mild adverse events on page 19, line 258 – page 20, line 265.

Comment 12: The authors should discuss factors such as the timing of infection relative to vaccination and circulating variants during the study period, and how this compares with infection rates in non-cancer populations.

Response: We discussed the possibility that the observed 27.6% infection rate may be related to the surge in the Delta variant during mid-2021 and the limited vaccine-induced immunity among cancer patients on page 20, lines 271 – 275.

Comment 13: The finding that patients under 60 report more adverse events is consistent with prior vaccine trials, but additional discussion on the biological or immunological rationale behind this observation would be valuable.

Response: The discussion is located on page 21, lines 286–294.

Comment 14: It would be helpful also to consider selection bias (e.g., which patients were reachable by phone) and the absence of objective immunogenicity assessments in the discussion.

Response: The discussion was added as suggested on page 21, line 301 and page 22, line 307.

Comment 15: Further discussion of the clinical implications, particularly regarding vaccine hesitancy and patient counseling.

Response: The discussion was emphasized on page 19, line 262 – page 20, line 265.

Comment 16: The authors should suggest prospective studies or multi-center collaborations that include immunogenicity and longer-term outcome measures.

Response: The suggestion was added to the conclusion on page 23, lines 325 – 329.

Comment 17: Suggest prospective multi-center studies for validation.

Response: We have revised the Conclusion to recommend future prospective, multi-center studies including immunogenicity assessments and longer-term outcome tracking to validate and extend our findings (page 23, lines 325 – 329).
