# Supplementary material for: Reported adverse events following COVID-19 vaccination in gynecologic cancer patients in Thailand: A descriptive study
Source: PLoS One. 2026 Feb 27;21(2):e0342303. doi: 10.1371/journal.pone.0342303 (PMC12948105; doi:10.1371/journal.pone.0342303)
Supplement: Attachment — Submitted filename: NP-Response_to_Reviewers_auresp_2.docx [file pone.0342303.s006.docx]

**Point-by-point Response to Reviewers**

**Response to Reviewer**

Comment 1: 1- Confounder control in logistic regression

Table 5 adjusts only for age and BMI; other clinically plausible covariates (comorbidities, ECOG, cancer stage, treatment status) were excluded despite their prevalence and potential to confound adverse-event reporting. Either present a formal rationale (e.g., events-per-variable calculation) for the reduced model or add a fully adjusted sensitivity analysis that includes the major covariates.

Response: We appreciate the reviewer’s insightful comment. In our primary logistic regression model, we included age and BMI as covariates because of their established biological plausibility and to preserve the stability of estimates given the limited number of adverse events relative to sample size. To address the reviewer’s concern, we have now conducted a fully adjusted sensitivity analysis that included additional clinically relevant covariates (comorbidities, cancer type, FIGO stage, ECOG performance status, and treatment status). The results of this sensitivity analysis are presented in Supplementary Table S2. The findings confirmed that only age <60 years remained significantly associated with the occurrence of any-grade adverse events after the first vaccine dose (adjusted OR 2.62, 95% CI 1.25–5.47, p = 0.010). Other covariates were not statistically significant, and the direction and magnitude of associations were consistent with the primary model, supporting the robustness of our main findings (Page17, Lines230-233).

2- Definition and ascertainment of adverse events

Adverse events were collected by telephone without objective confirmation; fever and other systemic symptoms relied on patient recall, supplemented by “Mohprompt” app entries. Provide the full interview questionnaire as Supplementary Material. Specify how grades (especially fever) were assigned when no temperature was recorded.

Response: We have now included the full structured interview questionnaire used to collect adverse event data as Supplementary Material (Supplementary File 3). Adverse events were graded according to the Common Terminology Criteria for Adverse Events (CTCAE) version 5.0. For fever, if patients did not recall the exact temperature, grading was based on the patient’s description of severity: (1) “low-grade or mild fever” was classified as Grade 1, (2) “moderate fever” interfering with daily activity as Grade 2, and (3) “severe fever requiring medical attention or hospitalization” as Grade 3. Other systemic symptoms (fatigue, myalgia, headache, etc.) were also graded using CTCAE v5.0 definitions based on interference with daily function, even in the absence of objective measurement. We have revised the Methods section accordingly (Page 7, Lines 121-128).

3- Interpretation of post-dose-2 infection rate

Seventy-eight of 283 patients (27.6%) developed PCR-confirmed COVID-19 ≥14 days after dose 2. This figure is interpreted without reference to background incidence. Add Thai incidence data for the corresponding period, or temper the interpretation and clearly acknowledge the absence of a comparator.

Response: We thank the reviewer for this important suggestion. Unfortunately, background national COVID-19 incidence data specific to our study population and time frame are not readily available for direct comparison. We have therefore tempered our interpretation and explicitly acknowledged the absence of a comparator in the revised Discussion section (Page 10, Lines 281-282 and Page 21, Line 292).
