# Supplementary material for: Reported adverse events following COVID-19 vaccination in gynecologic cancer patients in Thailand: A descriptive study
Source: PLoS One. 2026 Feb 27;21(2):e0342303. doi: 10.1371/journal.pone.0342303 (PMC12948105; doi:10.1371/journal.pone.0342303)
Supplement: Attachment — Submitted filename: V2Point-by-point.docx [file pone.0342303.s007.docx]

Point-by‑point response to reviewer

Comment 1

Due to a significant different proportion of Covid-19 vaccine uses during the phase of 1st&2nd doses (around 90% AZ), and 3rd&4th doses (around 99% mRNA), so AEFI from these two phases should not be compared together in general manner; different proportion of vaccine use should be addressed in the discussion, and also in the conclusion.

Response:

This comment has been fully addressed in both the Discussion and Conclusion.

Discussion section – new paragraph added

“Furthermore, direct comparisons of AEFI rates between primary (1st/2nd) and booster (3rd/4th) doses should be interpreted cautiously due to marked differences in vaccine platforms used. Approximately 87-93% of 1st and 2nd doses were AstraZeneca (viral vector), while 99% of 3rd/4th doses were mRNA-based (Pfizer/Moderna), reflecting Thailand's national vaccination rollout, which prioritized AZ early, followed by mRNA boosters. These platform differences may influence reactogenicity profiles, precluding general pooled comparisons across phases.” Line 323-330, Page 23.

Conclusion section – clarifying sentence added

“Although COVID‑19 vaccination was generally well tolerated across all doses, these findings should be interpreted with caution because the primary series consisted predominantly of AstraZeneca, while booster doses were almost exclusively mRNA vaccines, which may confound direct comparisons of adverse events following immunization between phases.” Line 359-364, Page 24-25.

Comment 2

Please give the detail for one patient who developed fever and seizure after 1st dose of AZ; was Vaccine-Induced Thrombotic Thrombocytopenia (VITT) was excluded? and what about the final clinical outcome of this patient.

Response:

We have expanded the Results to provide a clear description of this case, including explicit exclusion of VITT and the clinical outcome.

“This patient was diagnosed with encephalopathy attributed to stress or fever in the context of underlying pathologic brain changes from an old ischemic stroke. Vaccine-Induced Immune Thrombotic Thrombocytopenia (VITT) was systematically excluded through normal platelet count (>150 × 10⁹/L), absence of thrombosis on imaging (brain CT and venous Doppler), normal D-dimer, and negative anti-platelet factor 4, antibody enzyme-linked immunosorbent assay (ELISA), confirming non-thrombotic etiology per international diagnostic criteria. The patient fully recovered after supportive care (antipyretics, anticonvulsants, and neurology consultation) with no neurologic sequelae at 6-month follow-up.” Line 195-203, Page 12-13.

Comment 3

Table 5 - its title should indicate : Logistic regression...........after 1st dose of COVID-19 vaccine ( 93% AZ).

Response:

We have therefore revised the title of Table 5 to reflect the composition of the first‑dose cohort explicitly.

Revised Table 5 title:

“Table 5. Logistic regression of the association between demographics, clinical factors, and the occurrence of any‑grade adverse events after the first dose of COVID‑19 vaccine (92.9% AstraZeneca)” on Page 18.

Comment 4

Please give detail for abbreviations: FIGO, ECOG in the manuscript.

Response:

We have added full terms to the footnote of Table 1 on page 10:

“FIGO, International Federation of Gynecology and Obstetrics; ECOG, Eastern Cooperative Oncology Group performance status

Comment 5

Line 261 - should mention about the type of Covid 19 vaccines used in those studies; Ref 10-14.

Response:

The Discussion section referencing neurologic adverse events from the literature has been revised to specify the vaccine types associated with each cited report, based on the original articles.

The relevant sentence now reads:

“Several reports have described acute neurologic disorders, such as encephalopathy (hyperacute reversible following unspecified COVID-19 vaccine , post-Moderna), seizures (new-onset refractory status epilepticus following the ChAdOx1 nCoV‑19, AstraZeneca), acute disseminated encephalomyelitis (following various COVID-19 vaccination), neuroleptic malignant syndrome, and post-vaccine encephalitis, as secondary to the COVID-19 vaccine”. Line 268-274, page 20.
